# A Multiple Salient Features-Based User Identification across Social Media

**DOI:** 10.3390/e24040495

**Published:** 2022-04-01

**Authors:** Yating Qu, Huahong Ma, Honghai Wu, Kun Zhang, Kaikai Deng

**Affiliations:** 1 School of Automotive and Rail Transportation, Luoyang Polytechnic, Luoyang 471099, China; 18438550596@163.com (Y.Q.); zhangkun9595@163.com (K.Z.); 2 School of Information Engineering, Henan University of Science and Technology, Luoyang 471023, China; honghai2018@haust.edu.cn; 3 School of Computer Science (National Pilot Software Engineering School), Beijing University of Posts and Telecommunications, Beijing 100876, China; dkk@bupt.edu.cn

**Keywords:** across social media, user identification, salient features, similarity, bidirectional stable marriage matching

## Abstract

Identifying users across social media has practical applications in many research areas, such as user behavior prediction, commercial recommendation systems, and information retrieval. In this paper, we propose a multiple salient features-based user identification across social media (MSF-UI), which extracts and fuses the rich redundant features contained in user display name, network topology, and published content. According to the differences between users’ different features, a multi-module calculation method is used to obtain the similarity between various redundant features. Finally, the bidirectional stable marriage matching algorithm is used for user identification across social media. Experimental results show that: (1) Compared with single-attribute features, the multi-dimensional information generated by users is integrated to optimize the universality of user identification; (2) Compared with baseline methods such as ranking-based cross-matching (RCM) and random forest confirmation algorithm based on stable marriage matching (RFCA-SMM), this method can effectively improve precision rate, recall rate, and comprehensive evaluation index (F1).

## 1. Introduction

In recent years, social media such as Facebook has seen a rapid increase in user activity. According to statistics, 42% of users use multiple social media for information interaction at the same time [1]. For example, Sina Weibo is very similar to Twitter, and users can post updates instantly [2,3]. However, there is no direct connection between the existing major social media. Therefore, it is not easy for researchers to obtain a relatively complete user social graph. Identifying the multiple identities of users across social media is a way to solve this problem [2,4,5,6,7].

With the surge of social media users, the types of data generated have gradually diversified. The connection between different social media is not close enough, causing some malicious users to perform illegal actions by registering normal accounts, which seriously endangers network security [8]. In addition, a good network trust needs to be established between existing applications to improve users’ online experience. If a user can log in using an authorized method when logging in to a mobile application, it will greatly reduce the user’s login time and improve the user’s online experience. The realization of these applications requires the identification of the user’s major social media accounts to ensure that they can interconnect all the users’ social accounts. The realization of these needs is what motivates us to carry out user identification across social media.

User identification is also called anchor link. Many studies solve the problem of user identification by using user profile information. These profiles are mainly the information that users fill in when registering a personal account, such as user name, personal profile, interest, etc., [9,10,11,12,13,14,15]. Although this user information may be missing in the process of filling in, it can be filled in by relevant methods. Moreover, some user profile information plays an extremely important role in the process of user identification. Therefore, this type of user information can better complete the identification work. In addition, algorithms for user identification based on user network topology have also been widely used. The research ideas of this type of method mainly rely on the matching degree of the user’s friend relationship for user identification [16,17,18,19]. The similarity between users is determined by judging the similarity of nodes between users, but in practical applications, this type of method needs to be improved in terms of accuracy because of the heterogeneity among social media platforms. Finally, part of the research work is also based on user-published content for user identification, mainly using the behavior information generated by users of social media to analyze the entity users behind multiple virtual accounts. This type of method is less restrictive than the first two schemes, can be widely promoted, and has better recognition performance.

In view of the problems of the above different user identification methods [9,10,11,12,13,14,15,16,17,18,19], we conduct a fusion analysis of the user’s multi-dimensional information. The multiple salient features in the user’s display name, network topology, and user published content are extracted, and the characteristics of these three types of user information are analyzed. The relevant similarity calculation methods are then used for different feature types to obtain the similarity between user virtual account features. Moreover, the bidirectional stable marriage matching algorithm is used for the final matching of the multi-user accounts to be identified.

Our major contributions are summarized as follows:

- We extract and fuse multiple salient features contained in user display name, network topology, and published content.
- We adopt multi-module calculation methods to obtain the similarity between various redundant features.
- We design a bidirectional stable marriage matching algorithm to ensure that the different accounts achieve bidirectional optimality in the matching process in order to further improve user identification performance.
- We compare existing algorithms, and experiment results verify the effectiveness of the proposed algorithm.

The remainder of this paper is organized as follows. In Section 2, we introduce the related work. In Section 3, we summarize the problem definition. We review the user identification algorithm across social media in Section 4. Finally, we discuss the analysis of experimental results in Section 5 and conclude this paper in Section 6.

## 2. Related Work

User identification across social media has played an important role in many important research areas at present [6,7,20]. In this paper, we classify the existing work into three categories based on the different types of information used in the user identification process, i.e., user attribute information, user network topology information, and user behavior information.

### 2.1. User Attribute Information-Based User Identification

User identification algorithms based on user attribute information mainly use the basic information of the user for account identification. Raad et al. [21] designed a FOFA account matching strategy based on FOFA that can effectively achieve the identification of entities behind virtual accounts between different social media. Ye et al. [22] achieved user identification by calculating the similarity between user attribute information and using an objective method to weight the user attributes accordingly. Cortis et al. [23] assigned weights to various attributes of users and measured the similarity of various information from the semantic and grammatical levels to analyze whether different accounts are identical. Able et al. [24] aggregated user attribute information and users to be matched. Zamani et al. [25] focused on analyzing the user’s unit, interest, and other attribute information and combined the equal evaluation model and the complex training hybrid model to analyze and measure the similarity of multiple user attribute information and better realized the user identification. Xing et al. [6] proposed the use of multi-dimensional user attribute information for user identification, which involved 17 user attributes and achieved a better identification effect. However, from the perspective of user privacy protection [26], this work is susceptible to the limitations of the lack of user profile information. Moreover, the forgery of user attribute information has a greater impact on the final result of user identification. Therefore, the identification performance of this type of method needs to be improved.

### 2.2. User Network Topology Information-Based User Identification

Use identification algorithms based on user network topology information mainly rely on the friend relationship between users to determine the account identity. Relatively speaking, the user’s network topology is easy to obtain, and the probability of malicious forgery is small. Narayanan et al. [27] proved for the first time that user identification can be achieved by relying on user network topology information; however, the accuracy of their account matching is low. Bartunov et al. [28] integrated and analyzed user attribute information and network structure information and built an objective function. The final identification result was obtained by optimizing the objective function. Cui et al. [29] combined user attribute information and graph similarity to complete the mapping between different social media. Korula et al. [16] converted the specific problem of user account identification into a mathematical definition and explained that social media was obtained through the structural probability of the user’s social graph, and the formation of the edges of the social graph was also approximate. Tan et al. [17] modeled and analyzed the social relationships of users and mapped the associations between different users in social media to improve the accuracy of account matching. Li et al. [30] proposed a friend relationship-based network that recognizes the user’s identity by calculating the similarity of the nodes of the user’s friend network. However, there is heterogeneity among nodes in actual social media. Therefore, the identification performance of this type of method in the process of user identification will be affected.

### 2.3. User Behavior Information-Based User Identification

User identification algorithms based on user behavior information mainly analyze the content generated by users in social media to identify users. The user’s behavior is highly personalized and not easy to change [31], which can largely match the user’s own behavioral habits. Therefore, the use of related data mining algorithms to analyze these hidden behavior characteristics of users can greatly improve the identification performance of user identification. Goga et al. [12] analyzed and measured the geographic location, timestamp information generated by the user, and the user’s own writing habits to determine whether different user accounts are identical. Li et al. [15] used popular machine learning algorithms to solve the problem of user identification based on user behavior information.

In recent years, the development of mobile communication technology has made a great contribution of adding geographic coordinates when users publish their status. As the user’s motion trajectory is not easy to imitate, the application of geographic location information to user identification provides a new way to identify users across social media. Cao et al. [32] designed a user identification scheme for processing multi-source data by analyzing the co-occurrence frequency of user account trajectories in different social media. Hao et al. [33] proposed to equate the trajectory attributes generated by social users as a way to form a multi-grid structure sequence, which was subsequently vectorized using a vector transformation model, and the intersection values of trajectories of different user accounts were obtained using cosine similarity. Han et al. [34] converted each geographic coordinate point generated by the user into its corresponding semantic location word. The trajectory information generated by the user on the map had similar semantics. The authors then used LDA to form the user’s theme set and finally used the user trajectory. The frequency of co-occurrence is used to determine whether the account is identical. Chen et al. [35] achieved user identification across media by using a multi-graph convolutional network. In summary, this type of scheme breaks the limitations of the other two types of algorithms, so the identification algorithm based on user behavior information is a relatively good choice at present.

## 3. Problem Definition

This section defines the relevant terms used in this paper, integrates user display names, user network topology, and user behavior information to identify the entity users behind multiple social accounts, and details the process of user identification.

Social media refer to a virtual community platform for sharing and exchanging information between people. In social media, people can create basic information in accordance with the requirements of the system and use the interactivity of social media to communicate. It can be clearly seen from the above description that social media are mainly composed of three major components: basic user information, social information generated by users, and network structure diagrams formed by users. The content described above is defined below.

**Definition** **1** **(Social Media)**. *Given a social media SM = {V, P, N, C}, where V represents the user, P represents the basic information filled in by the user, N represents the user’s network friend relationship graph, and C represents the behavior information generated by the user.*

With in-depth analysis of the composition of social media user information, we can easily find that *P*, *N*, and *C* are all generated by *V*. To some extent, *P*, *N*, and *C* can be regarded as the attributes of *V*, which is the key to the study of social media. Therefore, user identification across social media has practical application value.

In this study, $SM_{A}$ and $SM_{B}$ are used to represent social media *A* and *B*, respectively.

**Definition** **2** **(Entity User)**. *Entity users are users who combine their profile information, behavioral information, and friend relationships.*

**Definition** **3** **(User Matched Pair)**. *Given social media $SM_{A}$ and $SM_{B}$, if social accounts $V_{Ai}$ and $V_{Bj}$ belong to the same person in real life, they then constitute a user matched pair $\left(V_{Ai},V_{Bj}\right)$.*

**Figure 1:**
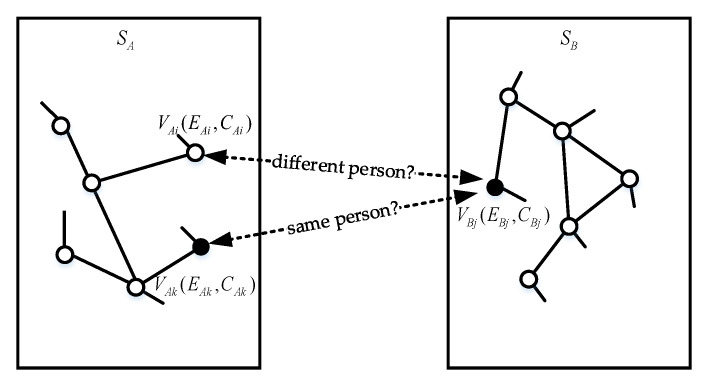
Example of user identification across social media. We need to determine whether accounts between different social media are the same offline entity.

In this section, we conduct user identification across social media based on the above three types of user information. In Figure 1, two social media $SM_{A}$ and $SM_{B}$ are given. Generally, there will be strong correlations between the same user accounts in different social media [13]. The work done in this paper is to explore the potential relationships between these accounts across social media. As can be seen from the figure, the focus of this research is to solve the following two problems:

(1) Given two accounts $V_{Ai}$ and $V_{Bj}$; can it be determined that these two accounts belong to the same person?
(2) Given two accounts $V_{Ak}$ and $V_{Bj}$; can it be determined that these two accounts belong to two different persons?

If the information sets of the two virtual accounts across social media are sufficiently similar, they are likely to be the same person. The more similar the redundant information they contain, the higher the probability that they belong to the same offline user. The user identification problem can be defined as:

(1) $$f_{Score}\left(V_{Ai},V_{Bj}\right)=\left\{\begin{array}{c} 1\;if\;V_{Ai}\;and\;V_{Bj}\;belong\;to\\ \;\;\;\;\;the\;same\;person;\\ 0\;otherwise. \end{array}\right.,$$

where $f_{Score}$ denotes the similarity score of $V_{Ai}$ and $V_{Bj}$.

For various reasons, some persons have multiple accounts in the same social media, but we usually think that these accounts are independent and belong to different persons. In other words, we only identify one of the accounts.

## 4. User Identification Algorithm across Social Media

### 4.1. Overall Framework of the Algorithm

In this paper, we mainly integrate the three prominent features of user display name, network topology, and behavior information to identify the entity users behind multiple social accounts. Due to the user’s own behavior habits and lazy psychology, the similarity probability of the display names filled out in multiple social media is very high. The friend relationship formed by users in social media is unique and can clearly map the relationship between accounts in different social media. Moreover, users can simultaneously post, share, and comment on real-time updates that interest them in major social media. Therefore, further research on user behavior information can effectively improve the overall identification performance.

**Figure 2:**
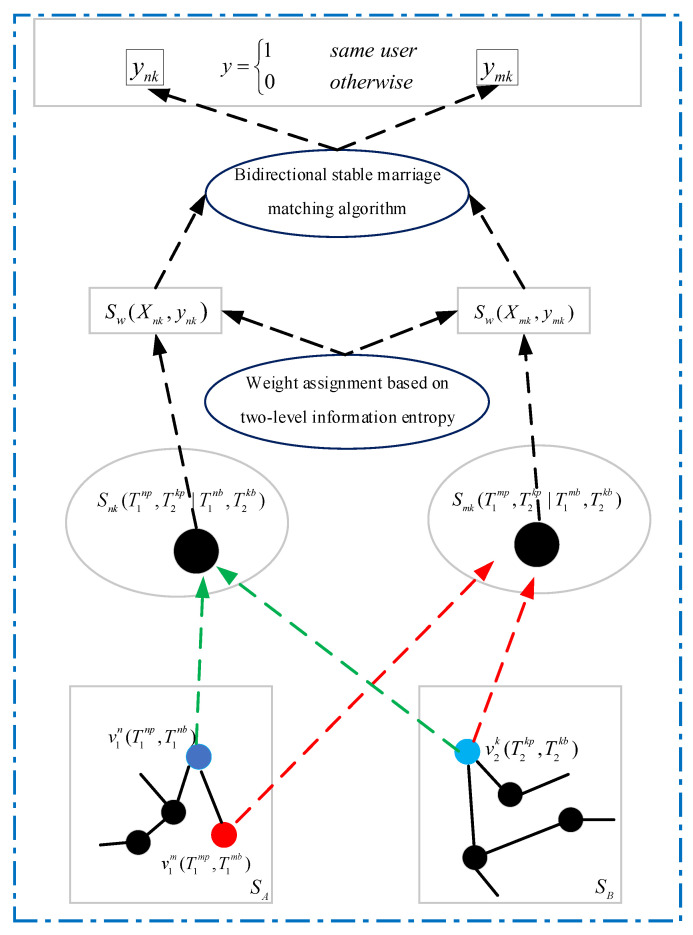
User identification across social media implementation framework. The three representative user information parameters uses different similarity calculation methods to achieve vectorized representation. Subsequently, we use the bidirectional stable marriage matching algorithm to obtain the internal associations between different social media accounts and output the final identification results.

As shown in Figure 2, we combine the three types of user information to extract user information redundancy in different types of information, and use different calculation methods to obtain similarity according to the nature of features. Specifically, given two social media *A* and *B*, and their corresponding three user accounts $v_{1}^{m}$, $v_{1}^{n}$, and $v_{2}^{k}$, where $v_{1}^{m},v_{1}^{n}\in S_{A}$, $v_{2}^{k}\in S_{B}$. The weight-based similarity corresponding to user account $v_{1}^{m}$, $v_{1}^{n}$, and $v_{2}^{k}$ are $S_{w}$. Taking user accounts $v_{1}^{n}$ and $v_{2}^{k}$ as an example, this pair of user accounts is mapped to node $S_{nk}$. Therefore, the identification problem of user accounts $v_{1}^{n}$ and $v_{2}^{k}$ is transformed into a classification problem. Next, we fuse and analyze the similarity of user’s multi-dimensional information, use the bidirectional stable marriage matching algorithm for user identification across social media, and output the final account matching results. That is, we need to determine whether $y_{nk}$ is equal to 1.

### 4.2. User Display Name Analysis

User display name can saliently reflect the daily psychological state and behavioral characteristics of users. We mainly analyze the salient features of user display name from three aspects. The display name is different from the frequently used username, which can change with the user’s habits and has a cyclical evolution. However, username is restrictive on some mainstream social media, i.e., they can only be a string of consecutive numbers, e.g., QQ, which makes the information less profitable for user matching. When users write display names in social media, there is a high probability that they will follow their previous wishes, which makes display names rich in redundant information. Therefore, better results can be achieved by using the redundant features contained in display names.

This paper mainly extracts and analyzes the length feature, character feature, and letter feature of the user’s display name to identify the user’s identity. When users register accounts in different social media, most of them will follow the above three features to write the display name. Li et al. [36] came to the conclusion that more than 45% of across social media users have the same display name. This research fully demonstrates the rationality of this work. The following will use different similarity calculation methods to measure and analyze the above three features.

#### 4.2.1. Length Feature

In the process of naming the display name of the user, the display names of the virtual accounts in different social media of the same user will have a certain similarity. The length ratio of the display names between different accounts is used to calculate the similarity of the length feature. The calculation formula is as follows:

(2) $$\begin{array}{c} S_{len}=\frac{\min(len\left(l_{1}\right),len\left(l_{2}\right))}{\max(len\left(l_{1}\right),len\left(l_{2}\right))},0\leq S_{len}\leq 1, \end{array}$$

where len(l) denotes the length of the display name. When $S_{len}$ is 1, it means that the display names of the accounts to be matched have the same length.

#### 4.2.2. Character Feature

The user’s display name in social media has the characteristics of characters. The longest common subsequence is used to obtain the similarity of character feature. The calculation formula is

(3) $$\begin{array}{c} S_{lcs}=\frac{len(lcs\left(n_{1},n_{2}\right))}{\min(len\left(n_{1},n_{2}\right))},0\leq S_{lcs}\leq 1, \end{array}$$

where $lcs(n_{1},n_{2})$ denotes the longest common substring of character strings $n_{1}$ and $n_{2}$, and $len(n_{1},n_{2})$ denotes the length of character strings $n_{1}$ and $n_{2}$.

#### 4.2.3. Letter Feature

The user’s display name is composed of the basic 26 English letters. The number of occurrences of the English letters is obtained to form a vector, and the cosine similarity is used for the calculation between the vectors as a way to obtain the similarity of the letter feature, which is calculated as follows:

(4) $$\begin{array}{c} S_{\cos\theta }=\frac{\sum_{i=1}^{n}A_{i}\times B_{i}}{\sqrt{\sum_{i=1}^{n}A_{i}^{2}}\times \sqrt{\sum_{i=1}^{n}B_{i}^{2}}}, \end{array}$$

where $A_{i}$ and $B_{i}$ denote the frequency of occurrence of the *i*th letter in the user’s display name.

### 4.3. User Network Topology Information Analysis

The user network topology information mainly analyzes the similarity of the friend relationship network formed by the user in social media to realize user identification. In the existing major social media, most users have a common phenomenon, namely "proximity", which is used to mine the correlation between nodes. Across social media, account matching work is then completed by quantifying the number of nodes to be shared by user nodes. Assume that two user nodes $v_{i}^{A}$ and $v_{j}^{B}$ are from social media *A* and *B*, respectively, and $\Gamma (v_{i}^{A})$ and $\Gamma (v_{j}^{B})$, respectively, represent the set of neighbor nodes shared by user nodes $v_{i}^{A}$ and $v_{j}^{B}$. Through research, there are many methods to measure the matching degree between user nodes. In comparison, the common neighbor index (CN) [37] is more commonly used and convenient to calculate, and its calculation formula is as follows:

(5) $$\begin{array}{c} S\left(v_{i}^{X},v_{j}^{Y}\right)=\left|\Gamma \left(v_{i}^{X}\right)\cap \Gamma \left(v_{j}^{Y}\right)\right|. \end{array}$$

### 4.4. User Behavior Information Analysis

User behavior information can also saliently reflect the daily behavior characteristics of users. The robustness of user identification can be further enhanced by measuring and analyzing the behavior information [2,6,11,15,19,36]. In this paper, the user behavior information is deeply mined on the basis of user display name and network topology structure. The following is a diversified analysis for different behavior features.

#### 4.4.1. Text Information

The behavior information generated by the user in social media can clearly map the user’s actual life characteristics. This paper mainly uses frequent pattern mining algorithms to obtain frequent items in text information and the support count corresponding to each frequent item and obtains the similarity of user text information by using frequent items and support counts. The calculation formula is

(6) $$\begin{array}{c} S_{ij}=\sum_{F_{n}\in i\cap j}[\left(1+C_{Ai}\left(F_{n}\right)\right)\times (1+C_{Bj}\left(F_{n}\right)){]}^{C_{F_{n}}}, \end{array}$$

where $C_{Ai}\left(F_{n}\right)$ and $C_{Bj}\left(F_{n}\right)$, respectively, represent the support counts of frequent items $F_{n}$ corresponding to users *i* and *j* to be matched in social media, and $C_{F_{n}}$ represents the number of item sets corresponding to $F_{n}$.

#### 4.4.2. Punctuation Mark

The use of punctuation marks in the process of users writing and posting content in social media reflects the daily writing habits of users from the side. Therefore, punctuation mark information is also an important indicator to measure user identity information. In order to calculate the similarity of punctuation marks between different user accounts, it is necessary to further quantify the punctuation marks of each user. Each dimension of the vector is $p_{i}=c_{i}/n$, where $c_{i}$ is the count of each punctuation mark, and *n* is the total number of blog posts. In this way, the punctuation vector corresponding to different user accounts can be obtained, and Formula (4) is used to obtain the similarity of different social accounts in this dimension.

#### 4.4.3. Status Timestamp

When offline entities post content across social media, their timestamps have a certain similarity, and this feature can reflect the daily active time of users. The similarity measurement of this feature is mainly determined by the ratio of the amount of information generated by the user in each timestamp to the total amount of information. First, it is necessary to divide the user’s day into 24 equal parts and count the number of dynamics published in each timestamp to calculate the average number of pieces of information in each timestamp. The whole calculation process obtains a 24-dimensional vector. Finally, the similarity measure for this feature can be measured using the state difference, which is calculated as

(7) $$\begin{array}{c} S_{st}=\sum_{t=1}^{24}\left|u_{it}-u_{jt}\right|, \end{array}$$

where $u_{it}$ and $u_{jt}$ denote the average number of dynamics of users *i* and *j* at time period *t*.

### 4.5. User Account Matching

After mining and computing the above user information, a similarity vector of three information fusions of social media users can be obtained. The obtained vector set is used as the input of the bidirectional stable marriage matching algorithm to achieve the purpose of user identification across social media. First, the overall similarity vector between different user accounts is used to construct the account matching score, and then the bidirectional stable marriage matching algorithm is used to identify the user to obtain the final identification result. The steps are as follows:

Step 1: The obtained matching scores are used to perform candidate matching on the user accounts to be matched in social media $SM_{A}$ and $SM_{B}$.

Step 2: According to the matching scores obtained during the account matching process, the matching scores of different accounts are sorted from high to low. The user in $SM_{A}$ needs to be matched with the user with the highest score in $SM_{B}$. If that account in $SM_{B}$ has not been previously matched with the remaining account in $SM_{A}$, it completes the match between that account and the account in $SM_{A}$ that is currently requesting a match. If the account is already a matched account pair, all accounts matching the account need to complete a scoring comparison process and output the account with the highest score that matches the account to form the final matching pair.

Step 3: If there are remaining user accounts in two social media that need to be matched, return to Step 2. The detailed account matching process of this algorithm is shown in Algorithm 1.
**Algorithm 1:** Bidirectional Stable Marriage Matching Algorithm.**Input:**$SM_{A}$ and $SM_{B}$.

**Output:** Final matching pairs $F_{match}$.
1:for each account $V_{Ai}\in SM_{A}$, the ranking of matching scores is named $\left\{V_{top-n}^{A}\right\}$2:for each account $V_{Bj}\in SM_{B}$, the ranking of matching scores is named $\left\{V_{top-n}^{B}\right\}$3:  Select account $V_{Bj}$ to be matched from $\left\{V_{top-n}^{A}\right\}$4:  if $V_{Bj}$ does not match5:    Add matching pair $\left(V_{Ai},V_{Bj}\right)$ to $F_{match}$6:    Set account $V_{Ai}$ and $V_{Bj}$ as a matching pair7:  else8:    Compare the matching scores of account $V_{Ai}$ and $V_{Ak}$ in $\left\{V_{top-n}^{B}\right\}$ (assuming that account $V_{Bj}$ has already been matched with account $V_{Ai}$)9:    if $V_{Ak}$ > $V_{Ai}$10:    Remove $\left(V_{Ai},V_{Bj}\right)$ from $F_{match}$11:    Add matching pair $\left(V_{Ak},V_{Bj}\right)$ to $F_{match}$12:    Set $V_{Ai}$ as the unmatched account13:    Set $V_{Ak}$ as the matched account14:  else: ignore $\left(V_{Ak},V_{Bj}\right)$15:return $F_{match}$


## 5. Analysis of Experimental Results

### 5.1. Acquisition of Datasets

When users apply for a new social media account, they will selectively associate their accounts in other social media for authorization. This phenomenon provides effective data support for scientific research. Google+ has this kind of data association phenomenon, and its user page will support authorization links so that users can be authorized when they log in. This paper uses this feature of Google+ to obtain user information on two social media with different functions, Facebook and Twitter, to verify the effect of the proposed algorithm in actual application scenarios. All experiments are performed on a computer with 32 GB memory and a 3.8 GHz CPU. We obtain a total of 13,473 and 12,945 user data from Facebook and Twitter, respectively, but after our screening, we finally select 6000 pairs of users who fit the experiment. The ratio between the training set and the test set is set to 3:1. Since there are still missing and noisy data incompatible with the data type in the user data, we can adopt appropriate filtering methods to supplement and replace.

**Figure 3:**
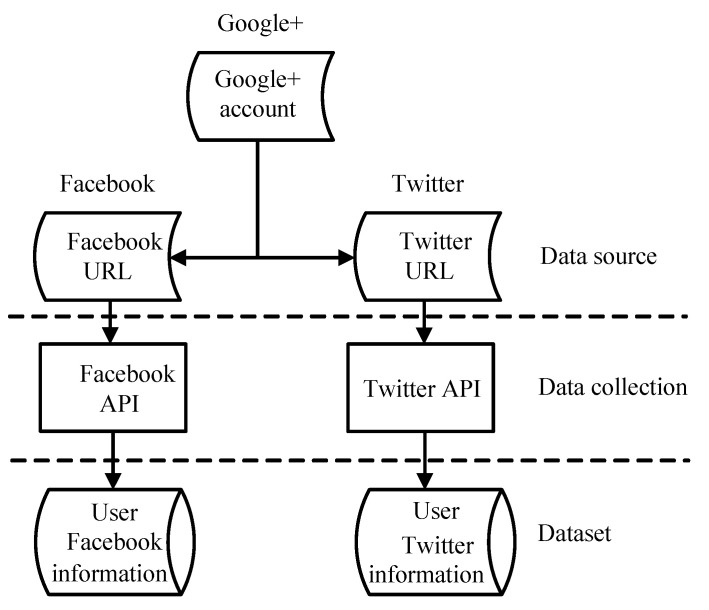
Dataset acquisition process. We obtain the ground truth of the user’s Facebook and Twitter accounts through partially open API.

The dataset required for the experiment in this paper mainly relies on an open dataset [38], and the way of obtaining user information is as described above. First, you need to determine whether there is a URL in the Google+ link; then, obtain the account of the corresponding user through a third party, and use the API interface to obtain the real dataset. Finally, the user data obtained through the link are imported into the database to provide data support for this experiment. The process of obtaining the experimental dataset is shown in Figure 3. We also present the specific data obtained in Table 1.

### 5.2. Evaluation Metrics

In the era of big data, due to different application scenarios, uniform evaluation metrics are needed to measure the effectiveness of user identification algorithms. Currently, the most widely used metrics are precision, recall, and overall evaluation metrics (F1), whose specific formulas are as follows:

(8) $$\begin{array}{c} Precision=\frac{tp}{\left(tp+fp\right)}, \end{array}$$

(9) $$\begin{array}{c} Recall=\frac{tp}{\left(tp+fn\right)}, \end{array}$$

(10) $$\begin{array}{c} F1=2\frac{precision\times recall}{\left(precision+recall\right)}, \end{array}$$

where tp denotes the number of account pairs that are correctly matched, fp denotes the number of acquired account pairs corresponding to entity users that are not homogeneous, and fn denotes the number of accounts that are not matched but are the same entity offline.

### 5.3. Experimental Results

#### 5.3.1. Ablation Study

**Figure 4:**
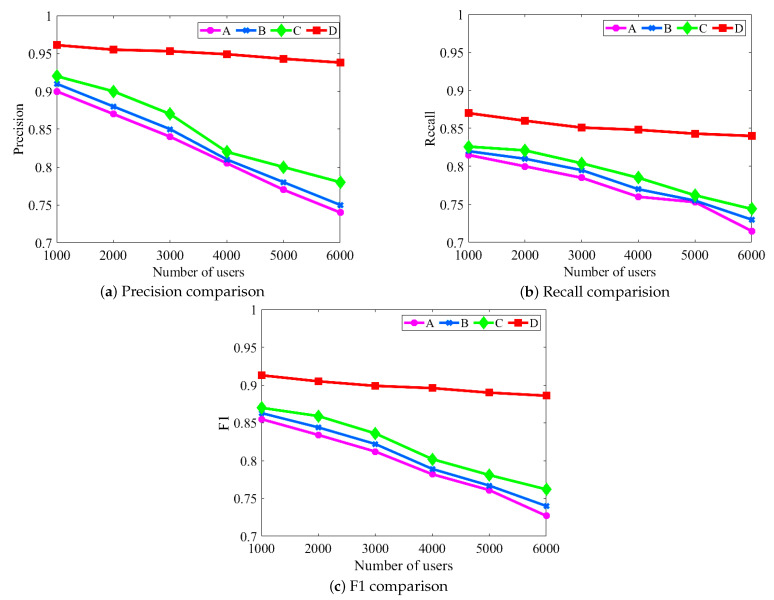
Comparison of single user information identification performance. We can see that multi-information fusion for user identification across social media can achieve better information complementarity. Note that due to the contribution of different user information, different types of information also have differences in the results of user identification.

During the experiments, the three types of user information used in this paper were first tested individually, and the effects of user display names, network topology information, and behavioral information on user identification were analyzed. The experimental results are shown in Figure 4. In this paper, the performance of the above three types of user information individually is compared with the fusion analysis of user information to illustrate the effectiveness of the research work. In order to show the effect more clearly in the figure, we define A, B, C, and D to characterize the above classification.

- A represents the performance curve based only on the user’s display name.
- B represents a performance curve based only on the user’s network topology.
- C represents a performance curve based only on user behavior information.
- D represents a performance curve based on multiple salient features.

From Figure 4, it can be seen that the performance of all four schemes tends to decrease as the number of matched pairs increases; however, it can be seen that the rate of decrease of scheme D is flatter compared to the other three schemes. This is because solution D fuses and analyzes the multi-dimensional information of users and has better universality. We observe that scheme D is improved by 15.98%, 14.49%, and 13.31% in terms of precision compared to A, B, and C, respectively. For recall, we observe that scenario D improves 13.23%, 12.14%, and 10.01% compared to A, B, and C, respectively. Similarly, for F1, we observe that scenario D improves 14.23%, 13.14%, and 11.78% compared to A, B, and C, respectively.

In contrast, scheme A increases the similarity of display names between user accounts substantially as the number of users increases, which makes it more difficult for user identification. When the number of users increases in the B scheme, some users will have the characteristics of sparse friend relationships, which makes it difficult to identify the user, so that the overall performance is reduced. Moreover, the rate of decline of C scheme is better than that of A and B schemes, because C scheme is not restricted by A and B schemes. As the number of users increases, the content posted by users is largely unaffected. Based on the above analysis, the effectiveness of the scheme proposed in this paper can be obtained. In addition, as shown in Table 2, to observe the matching between users more intuitively, we enumerate the matching results between different information of a pair of users, which further demonstrates the effectiveness of the proposed algorithm.

#### 5.3.2. Baseline Comparison

This research proposes a user identification across social media algorithm based on multiple salient features. A comparative analysis with two baselines, namely RFCA-SMM [39] and RCM [40], was completed. The reasons why we compare these two baselines are as follows. First, RFCA-SMM analyzed 20 user information items for the account matching across social media, which involved too much user information. Due to the privacy issues in existing social media, we chose RFCA-SMM for comparison to show whether our algorithm has good performance compared to RFCA-SMM based on a small amount of user information. Second, we compared with RCM mainly to illustrate the effectiveness of the matching algorithm. MSF-UI performs reverse confirmation of matching accounts, which is not available in RCM, so that the matching error rate can be effectively reduced.

- RFCA-SMM is a random forest secondary confirmation algorithm based on stable marriage matching. The stable marriage matching algorithm obtains candidate matching pairs of multi-user accounts and then trains the random forest model to perform secondary confirmation on the obtained candidate matching pairs, thereby further improving the precision of multi-user account matching.
- RCM uses account attribute similarity and user surrounding score, and then selects the user with the highest current score as the candidate matching user. Subsequently, the concept of user matching score (UMS) is proposed. UMS is combined with attribute similarity and network structure, and the user who matches the candidate user is determined based on this score.

Whereafter, we conduct a comparative analysis to verify the improvement of the proposed algorithms in this study in terms of various evaluation metrics. RFCA-SMM analyzes 20 items of user information and weights the user information, but there is falsification of user profile information. The results of RCM in the process of user identification are largely governed by the seed nodes.

**Figure 5:**
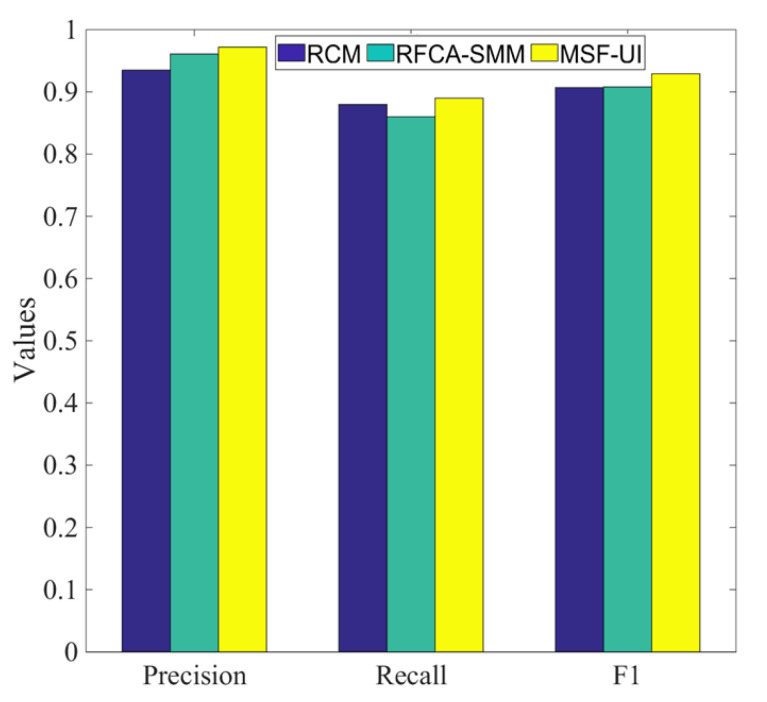
Comparison of identification performance of various algorithms. Since MSF-UI focuses on specific user information; it fully considers the contribution of different information in the process of user identification. MSF-UI achieves the effect of high accuracy rate with less user information. Note that MSF-UI effectively protects user privacy.

It can be seen from Figure 5 that MSF-UI is superior to RFCA-SMM and RCM in terms of precision, recall, and F1. MSF-UI combines the three types of user information. Compared with RFCA-SMM, MSF-UI greatly reduces the amount of user information used and effectively protects user privacy. Compared with RCM, MSF-UI is not restricted by seed nodes, which is of great significance for improving the robustness of user identification. In order to more clearly characterize the effectiveness of MSF-UI, it can be seen from the comparison of evaluation indicators of user identification in Figure 5 that in terms of precision, MSF-UI is higher than RCM and RFCA-SMM by 3.7% and 1.1%, respectively. In terms of recall rate, MSF-UI is 1% and 3% higher than RCM and RFCA-SMM, respectively. Moreover, in terms of F1, MSF-UI improved this metric by 2.2% and 2.1% compared to RCM and RFCA-SMM, respectively. Through the above analysis, the algorithm proposed in this paper will get better identification results in practical applications, which fully demonstrates the effectiveness of MSF-UI. Furthermore, to better demonstrate the progressiveness of our work, we also compare with our newly published work [2].

The focus of [2] is mainly the in-depth mining of user friend networks, whereas our current work focuses on the analysis of multiple salient features of user behavior habits and uses the user friend networks as a feature to enhance the identification performance. The results show that MSF-UI is 2.98%, 1.87%, and 2.16% higher than [2] in precision, recall, and F1, respectively.

Note that the domain of applications designed for across social media user identification is relatively sensitive, and it is related to the privacy and security of users. Therefore, the identification precision is very demanding. Compared with the baselines, the precision of the work in this paper is improved by an average of 2%. This has great practical significance among large-scale social media users and can greatly improve the quality of user experience.

#### 5.3.3. Complexity Analysis

Generally speaking, the best matching result for across social media user identification is to satisfy one-to-one constraints. The complexity of our proposed Algorithm 1 is $o(Nn^{2})$. We compare the other two baselines, where the complexity of RFCA-SMM is $o(Kn^{2})$, and the complexity of RCM is $o(Mn^{2})$, where K>M>N; *K*, *M*, and *N* represent the number of dimensions used by the algorithm in user identification.By comparing our algorithm with baselines, which can prove the effectiveness and rationality of MSF-UI.

## 6. Discussion and Future Directions

There are still many shortcomings in the existing user identification across social media algorithms. More in-depth research in this area needs to be conducted. Therefore, future research work will continue to explore, in combination with the latest progress of some research, the following five aspects:

- There are many different types of user information in social media, and some of them have not been deeply mined and analyzed [41]. For some existing special institutions, they need to be highly recognized in terms of user identification. At this time, only a few dimensions of user information can be integrated and analyzed to have certain limitations on the accuracy of user identification.
- When the model identifies large-scale users, the identification performance of existing algorithms will gradually decrease, which is also reflected in the experimental part of this paper. Community discovery in a complex network can solve this problem well [35]. By dividing a large-scale dataset into several communities and identifying between communities, it can effectively alleviate the problem that the existing identification algorithm reduces the identification performance as the dataset increases.
- Existing identification algorithms only pay attention to the user’s neighbor nodes, that is, single-hop nodes, while ignoring the role of multi-hop nodes in the friend relationship when analyzing the user’s network topology [30]. Therefore, in future research work, the contribution of multi-hop nodes to multi-user identification can be deeply analyzed, so that the performance of existing identification algorithms based on network topology can be further improved.
- Although a small amount of user information is used to achieve better multi-user identification, it also provides a shortcut for some malicious attackers in the network to obtain normal user information [26]. Therefore, future work should consider from the perspective of game theory to balance the relationship between user information and privacy protection.
- As supervised learning methods rely on pre-matched user pairs, which are difficult to obtain, the classification models adopted in the existing work lack sufficient training sets, which may lead to a lack of accuracy of the classifier, so obtaining more valid data and constructing more suitable classification models will further improve the accuracy of identification.

## 7. Conclusions

In the era of big data, the channels for obtaining user information have become more abundant. Identifying users in social media can benefit network security, information retrieval, and user recommendation. When identifying users in social media, users’ private information should also be fully considered. We propose a user identification across social media algorithm based on MSF-UI, which further improves the universality of the identification algorithm by analyzing the redundant features contained in users’ display names, using diverse computational methods to measure their similarity depending on the features, and fusing and analyzing information about users’ network topology to exploit their friendships. Moreover, the algorithm also fully considers the user’s personalized behavior habits, starting from multiple dimensions and improving the comprehensive performance of user identification. Finally, the bidirectional stable marriage matching algorithm is used to determine the identity of the account pairing. Experimental results show that the algorithm proposed in this paper greatly improves the identification performance and effectively protects user privacy. We should use less user information to identify users to avoid privacy issues in future work. Meanwhile, our algorithm is inevitably affected by the size of the dataset.

## Figures and Tables

**Table 1:** User data type classification.

User Data	Data Type
Length feature	
Character feature	User display name information
Letter feature	
Friend relationship	User network topology information
Text information	
Punctuation mark	User behavior information
Status timestamp	

**Table 2:** Illustration of matching results by different social media users.

User Information	Matching Metrics	Matching Thresholds
Length feature	0.98	0.9
Character feature	0.87	0.8
Letter feature	0.98	0.92
Friend relationship	23	20
Text information	5500	5000
Punctuation mark	0.98	0.9
Status timestamp	0.68	0.6

## Data Availability

Not applicable.
